# Estimation of mass loss under wear test of nanoclay-epoxy nanocomposite using response surface methodology and artificial neural networks

**DOI:** 10.1038/s41598-025-05263-y

**Published:** 2025-06-06

**Authors:** Manjunath Shettar, Ashwini Bhat, Nagaraj N. Katagi

**Affiliations:** 1 Department of Mechanical and Industrial Engineering, Manipal Institute of Technology, Manipal Academy of Higher Education, Manipal, 576104 Karnataka India; 2 Department of Mathematics, Manipal Institute of Technology, Manipal Academy of Higher Education, Manipal, 576104 Karnataka India

**Keywords:** Nanoclay, Epoxy, Composite materials, Composite manufacturing, Wear, Response surface methodology, Artificial neural networks, Scanning electron microscopy, Materials science, Mechanical engineering

## Abstract

In this work, the wear behavior of nanoclay-epoxy nanocomposites is studied through Response Surface Methodology (RSM) and Artificial Neural Networks (ANN) as predictive models. This study aims to measure mass loss under wear conditions by studying critical parameters like nanoclay wt%, load, speed, time, and water soaking time. Experimental runs are planned based on the Box-Behnken design of RSM to create a regression model, which is then validated by ANOVA analysis. An ANN model is also trained and tested to improve predictive accuracy, performing better than RSM. The results show that wear resistance is greatly enhanced by increasing nanoclay content, which minimizes material loss. Water absorption adversely affects wear performance, resulting in enhanced mass loss caused by plasticization and swelling. The ANN model is more accurate in prediction than RSM, with minimal variation from experimental data. Scanning Electron Microscopy (SEM) analysis gives insights into wear mechanisms. The research demonstrates the efficiency of combining statistical and machine-learning methods for optimizing wear-resistant polymer nanocomposites.

## Introduction

Epoxy resins are commonly used in numerous engineering applications owing to their good mechanical properties, chemical resistance, and thermal stability. These thermosetting polymers have high adhesion strength, low shrinkage, and excellent electrical insulation, which makes them suitable for coatings, adhesives, and structural composites^1^. Nevertheless, despite their benefits, epoxy-based materials usually experience wear resistance problems, especially in applications exposed to friction and mechanical stresses^2^. The brittleness inherent in epoxy and its low toughness cause material degradation, decreasing the component’s life and performance when it is subjected to wear conditions^3^. Wear is a process of material degradation that results from the surfaces in contact experiencing frictional stresses, causing material loss in terms of mass reduction. Nanoparticles are added to improve the wear characteristics of epoxy. These nanoscale reinforcements enhance mechanical strength, thermal stability, and tribological properties by limiting polymer chain mobility, load distribution, and hardening^4,5^. This enhancement decreases material removal, reducing the mass loss during wear conditions^6^.

Several studies have demonstrated that incorporating nanoparticles such as alumina $\:(Al_2O_3)$, tungsten carbide (WC), titanium dioxide $\:(TiO_2)$, and boron can significantly enhance wear resistance. Alhazmi et al.^7^ investigated the influence of nano-$\:Al_2O_3$ addition on the wear resistance of epoxy composites. The wear rate reduces after incorporating nanoparticles, and 2 wt% of $\:Al_2O_3$ provides optimal wear resistance.​ This is attributed to $\:Al_2O_3$‘s ability to form a protective film on the epoxy surface, reducing material loss. Similarly, Al Saadi et al.^8^ studied the effect of incorporating tungsten carbide (WC) nanoparticles on the wear rate of epoxy composites. The study demonstrates that adding WC content enhances wear resistance by reducing the wear rate, with optimal results at 2% WC loading. The hard outer layer formed by WC particles helps protect the surface from wear, reducing material loss. Apay and Kilinçel^9^ investigated the wear resistance of epoxy composites reinforced with nano-sized $\:Al_2O_3,\:TiO_2,\:$and boron particles. Results indicate that adding nanoparticles significantly improves wear resistance, with boron-reinforced epoxy composites exhibiting the highest wear resistance. The friction coefficient of epoxy decreases from $\:0.4\:-\:0.6$ to $\:0.2\:-\:0.4$ with the inclusion of nano boron, reducing surface roughness from $\:1.4\:\mu\:m$ to $\:0.32\:\mu\:m$. Pin-on-disc wear tests confirm that 1% boron addition yielded the lowest wear rates, demonstrating superior wear performance compared to $\:Al_2O_3$ and $\:TiO_2$ reinforcements​.

Nanoclay has emerged as a promising reinforcement among various nanoparticles due to its solid lubricating effect and ability to form a transfer film that reduces friction and surface degradation. Kini et al.^10^ investigated the impact of different wt.$\:\%$ of nanoclay addition on the wear property (mass loss (mg)) of nanoclay-polyester nanocomposites. The wear tests are conducted under a controlled environment with fixed speed (200 rpm), load (2 kg), and duration (10 min). Pure polyester exhibits the highest mass loss of 159 mg, whereas 2 and 4 wt% nanoclay addition declines mass loss to 132 and 95 mg, respectively.

Wear properties are influenced by multiple factors, including material composition, aging type, applied load, speed, and duration. Investigating wear behavior across different levels of these factors requires numerous experimental trials, which can be resource-intensive in terms of materials, time, energy, and cost^11^. The design of experiments (DOE) approach helps minimize the number of trials while maintaining the reliability of the results. RSM is an effective technique for developing predictive models that account for multiple factors and their interactions^12,13^. Researchers have widely used RSM to enhance and optimize the characteristics of various composite materials.

Studies by Avalappa et al.^14^ applied RSM (Box–Behnken design) to optimize wear performance of 3D-printed PLA–graphene composites using fused deposition modeling. RSM is utilized to develop predictive models for surface roughness and wear loss, evaluating the influence of layer height, printing temperature, and printing speed. The desirability function approach, an RSM-based multi-objective optimization method, is applied to determine the optimal parameter settings, minimizing both surface roughness and wear loss. Sinha et al.^15^ employed RSM to analyze and optimize the sliding wear behavior of hybrid abaca–epoxy composites. The study utilizes a full factorial experimental design to develop a quadratic regression model, correlating the sliding wear response with input factors—abaca fiber weight%, red mud weight%, and red mud particle size. The developed RSM model is validated using ANOVA, demonstrating a high R² value of 95.28%, confirming its accuracy in predicting wear behavior. The model is further optimized, identifying the most favorable composite formulation for minimal wear loss​. Shettar et al.^16^ successfully applied RSM to analyze the wear properties of polyester-nanoclay nanocomposites. RSM’s “central composite face-cantered” design is used to analyse the role of nanoclay content, load, speed, and time on wear behavior. The investigation successfully proves the improvement in the performance of polyester due to the reinforcement of nanoclay and that RSM is a sturdy tool for the optimization of wear behavior with minimal testing.

Besides statistical methods, computational modeling techniques like Artificial Neural Networks (ANNs) have become increasingly popular for forecasting material properties. ANNs show better learning abilities than conventional regression models and can establish intricate nonlinear associations in wear behavior^17^. In predictive accuracy, ANN models tend to outperform Adaptive Neuro-Fuzzy Inference System (ANFIS) models. Most of the research has been concerned with selecting ANN hyperparameters from experimental data and particular applications, emphasizing the necessity of automatic optimization. Metaheuristic algorithms are commonly used for parameter tuning in many fields.

Recent research has shown the potential of ANN in wear prediction and the fact that it usually surpasses empirical models in accuracy^18^. Thimmaiah et al.^19^ used ANN to predict wear behavior of Kenaf–Kevlar fabric-reinforced hybrid polyester composites. The research adequately proves ANN’s ability to enhance wear resistance predictions and provides valuable information about composite material performance. Suresh et al.^20^ also improved ANN predictive ability by incorporating the Cuckoo Search-Optimized Artificial Neural Network (CS-ANN) model with high correlation with experimental results. Incorporating the Cuckoo Search Algorithm improves ANN hyperparameters, resulting in an optimally trained model with negligible error. The research successfully validates ANN’s efficacy in predicting wear behavior and provides a consistent method for material property analysis. Ho et al.^21^ proposed an ANN model for predicting Young’s modulus of polymer/carbon nanotube (CNT) composites, overcoming the shortcomings of conventional experimental and numerical approaches. The ANN model is optimized by parametric analysis and exhibits high accuracy with correlation coefficients of 0.986 for training and 0.978 for testing. The research verifies ANN’s capability to predict the mechanical properties of nanocomposites efficiently, saving computational time and experimental expenses.

The integration of RSM and ANN provides an effective method of optimizing and predicting the wear behavior of polymer composites. Arunachalam et al.^22^ deliberated the mechanical properties of jute/kenaf/glass fiber composites with multi-walled carbon nanotubes (MWCNTs) reinforcement, using RSM and ANN to optimize. RSM is utilized to study the combined influence of fiber orientation, sequencing, and MWCNT content on hardness and flexural strength. The ANN model shows excellent accuracy (95%) in identifying the mechanical properties, which is better than regression-based techniques. The work successfully couples RSM and ANN to optimize performance with a powerful approach to composite material design. Ladaci et al.^23^ investigated the water uptake behavior of recycled high-density polyethylene (RHDPE) biocomposites reinforced with treated palm waste fibers, utilizing both RSM and ANN for predictive modeling. RSM is applied to optimize fiber content and immersion duration, while ANN demonstrated superior predictive accuracy, with correlation coefficients above 0.97. Results indicate that fiber content and immersion time significantly influence water uptake, with ANN outperforming RSM in prediction reliability. The study effectively showcases the integration of ANN and RSM for optimizing biocomposite performance, providing valuable insights for material engineering applications.

Despite the extensive literature on polymer composites, limited studies have explored the combined use of RSM and ANN to optimize wear properties. Moreover, to the best of the authors’ knowledge, no work has been reported on the study of the influence of parameters viz., nanoclay (wt%), load (kg), speed (rpm), time (min), and water soaking (days), on wear property (mass loss) by applying RSM and ANN model for wear prediction.

This study aims to experimentally investigate the wear performance (mass loss) of the nanoclay-epoxy nanocomposite, focusing on mass loss as the primary response variable. The Box-Behnken design of RSM is utilized to model the effects of key process parameters (nanoclay, load, speed, time, water soaking) on the mass loss under the wear test of the nanoclay-epoxy nanocomposite. An ANN model is also developed to predict the wear behavior based on experimental data, providing a comparative analysis of statistical and machine learning approaches. Furthermore, scanning electron microscopy analysis is performed to correlate the wear mechanisms with material microstructure, offering insights into the advancement of wear-resistant polymer nanocomposites by providing a systematic framework for predictive modeling and optimization.

## Methodology

### Materials

Atul Polymers in Gujarat, India, supplies epoxy resin (L-12) and hardener (K-6) at a 10:1 mixing ratio. Sigma Aldrich provides a surface-modified nanoclay. Table 1 lists a few properties of the materials selected for the study.

**Table 1:** Selected material properties.

Sl No	Material Name	Properties
1	Epoxy resin (L-12) and hardener (K-6)	Tensile strength (MPa) - 55 - 70
		Tensile modulus (GPa) - 2.5 - 04
		Flexural strength (MPa) - 120 - 140
		Density (g/cm^3^) - 1.15
2	Nanoclay (Montmorillonite (MMT))	Appearance (color) - White to off-white
		Appearance (form) - Powder
		Size - < 20 μm
		Bulk density - 200–500 kg/m^3^
		Surface modified contains 15–35 wt%
		octadecylamine, 0.5–5 wt%
		aminopropyltriethoxysilane.

### Composite preparation

Initially, nanoclay is mixed with epoxy through mechanical stirring for 120 min to achieve uniform distribution. This is followed by sonication for 30 min to further enhance nanoclay dispersion within the epoxy matrix. The mixture is placed in a vacuum chamber for 30 min to remove the entrapped air bubbles. Following degassing, the K-6 hardener is thoroughly combined with the nanoclay-epoxy mixture and homogenized using a spatula. The final mix is then poured into pre-fabricated molds for curing.

### Wear test

**Fig. 1:**
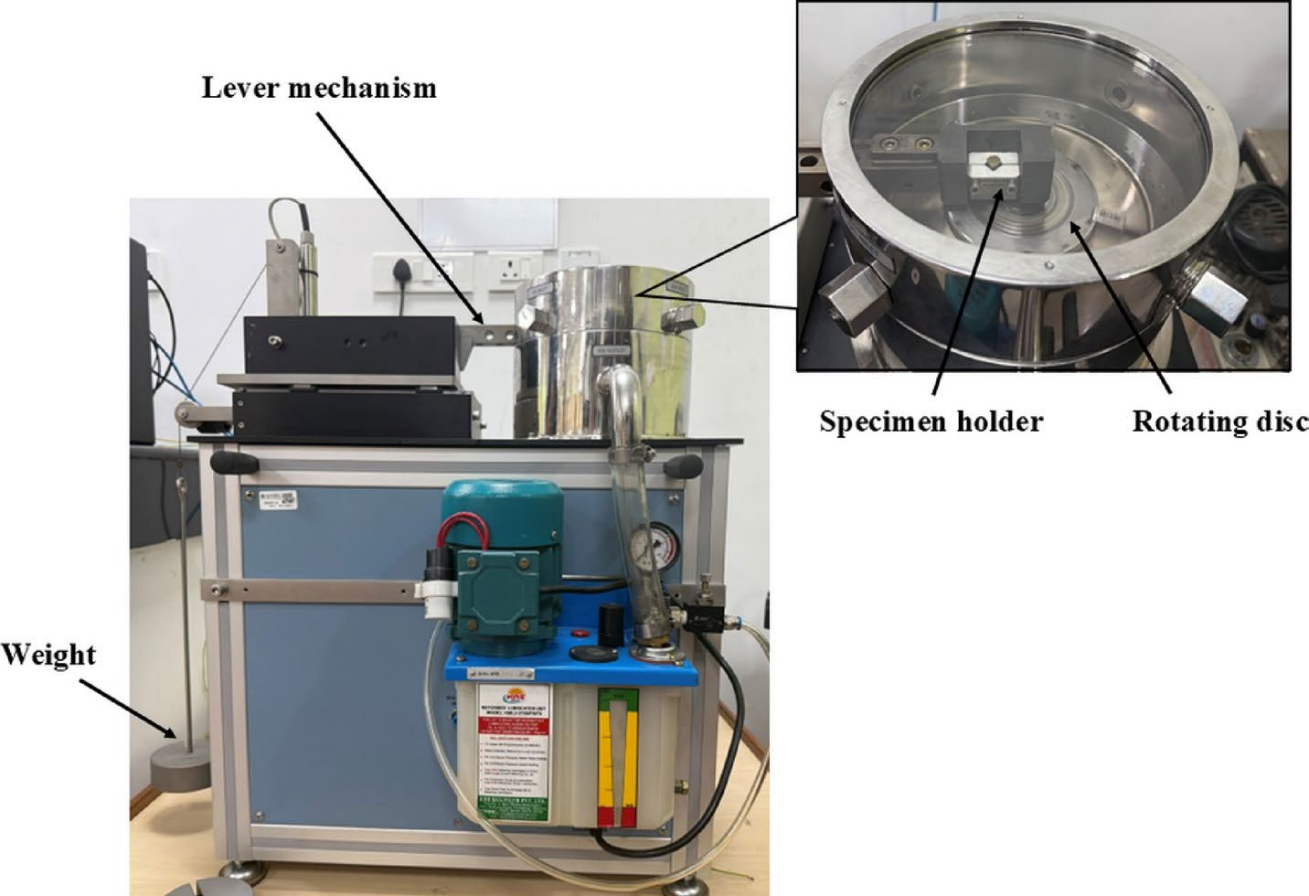
Pin-on-disc tribometer set up.

The wear test of the specimens is performed using a pin-on-disc tribometer (Fig. 1) following ASTM standard G-99^24^. The machine features a hardened EN 31 steel disc with a roughness of Ra = 5 μm. The test sample is securely mounted on the disc using a holder with four screw fasteners, ensuring perpendicular alignment to the rotating disc. The applied load is controlled through a lever mechanism to maintain consistent contact pressure. Test parameters, including rotation time, disc speed, and track diameter, are manually adjusted for each test iteration to ensure precise control over wear conditions. Each specimen’s mass loss under the wear test is determined by measuring its weight before and after the test.

### Design of experiments

The Box-Behnken design of RSM is employed to minimize the number of required trials, reduce cost, and save time while ensuring robust statistical analysis^25^. The methodology enables the evaluation of both individual and interaction effects of various factors (at different levels) on mass loss (wear property). RSM is employed to determine the optimal solution by developing a predictive function for mass loss (response variable) and analyzing the influence of key parameters viz., nanoclay (wt%), load (kg), speed (rpm), time (min), and water soaking (days).

Minitab Statistical Software Version 22.1.0 (https://www.minitab.com/en-us/) is utilized to create the matrix design and analyze the experimental data. Three levels for each factor, viz., nanoclay, load, speed, time, and water soaking, are considered, as shown in Table 2.

**Table 2:** Different factors with varying levels used in the study.

Factors	Levels		
	Low (− 1)	Middle (0)	High (+ 1)
Nanoclay (wt%)	0	3	6
Load (kg)	1	2	3
Speed (rpm)	150	200	250
Time (min)	10	15	20
Water soaking (days)	0	30	60

### Artificial neural network

This methodology involves creating and training an ANN to predict a target variable based on several input variables. The ANN model utilized in this study is a feedforward neural network designed to predict mass loss under wear conditions. The architecture consists of an input layer with five neurons corresponding to nanoclay wt%, load, speed, time, and water soaking duration; two hidden layers with ten neurons each, employing hyperbolic tangent sigmoid activation functions; and an output layer with a single neuron representing mass loss, using a linear activation function.

**Fig. 2:**
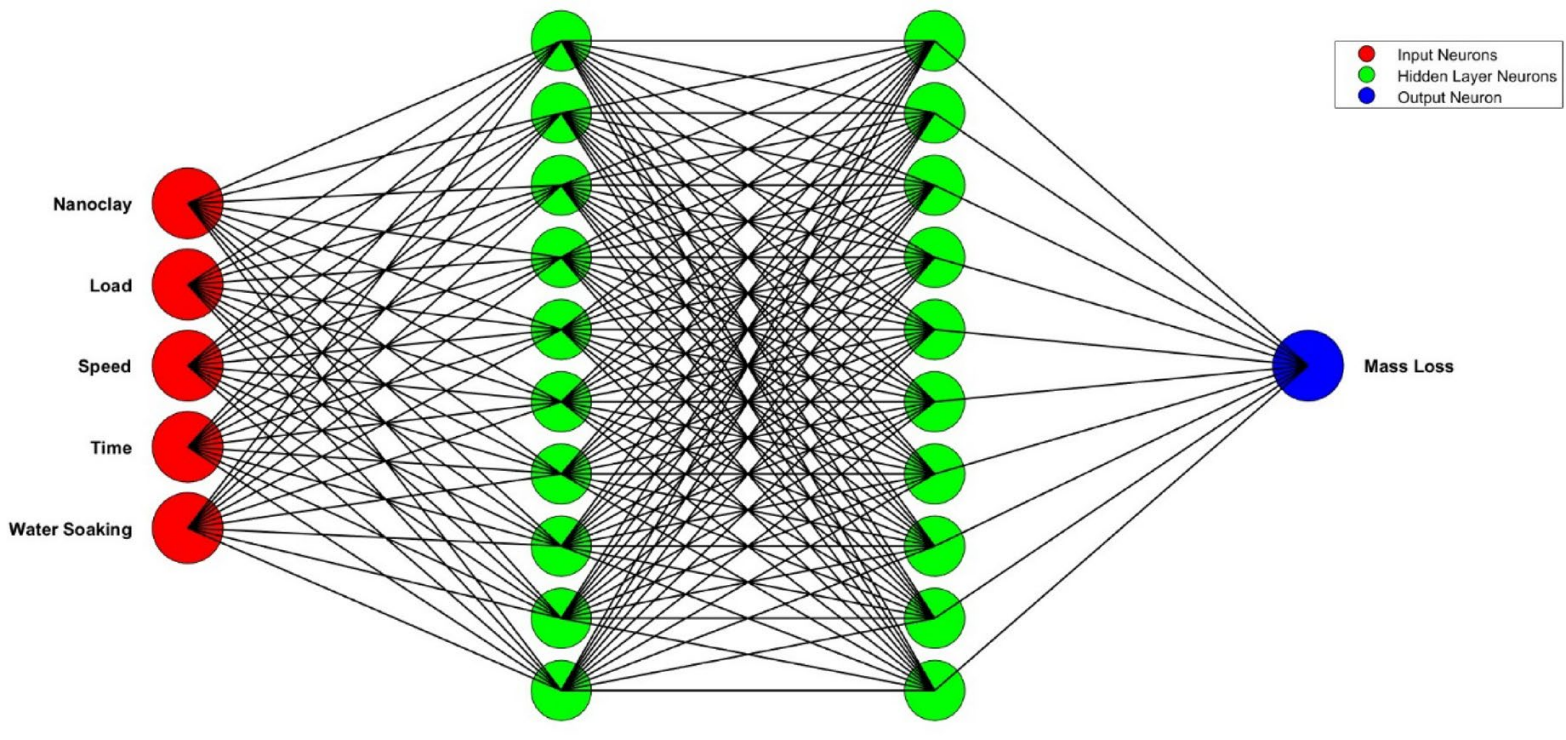
ANN architecture.

Data processing is performed by normalizing all input parameters using min-max scaling to ensure balanced feature contributions during training. The data set is then split into training (80%) and testing (20%) subsets. The network is trained using the Levenberg-Marquardt backpropagation algorithm^26,27^, chosen for its fast convergence and suitability for small to medium-sized datasets. Post-training, the ANN model generates the mass loss predictions, which are subsequently denormalized to their original scale. In this study, MATLAB is leveraged to optimize a feedforward neural network for regression, and model accuracy is evaluated using performance metrics such as Mean Squared Error (MSE) and R-squared ($\:{R}^{2}$), ensuring reliable predictive capability for wear analysis. Figure 2 displays the ANN architecture used in the present study.

### Morphology analysis

The surface of the worn-out specimen is examined using a “scanning electron microscope” (ZEISS, Model: EVO18). Samples are cut to the required size to fit into the SEM sample holder. To ensure effective imaging, the sample surface needed to be electrically conductive. The samples are electrically grounded to prevent electrostatic charge accumulation during electron irradiation. A sputtering machine applies a thin layer of a conductive material, such as 80:20 gold-palladium, to the sample surface.

## Results and discussion

### Response surface methodology

#### Box-Behnken design

Table 3 displays the Box-Behnken design, and 46 tests are conducted in total. The response variable for this current analysis is mass loss (mg).

**Table 3:** Box–Behnken experimental design with levels, factors, and response variable.

Nanoclay (wt%)	Load (kg)	Speed (rpm)	Time (min)	Water soaking (days)	Mass loss (mg)
0	2	250	15	30	165
3	2	200	10	0	69
3	2	250	20	30	114
0	2	200	10	30	135
3	2	200	15	30	90
3	2	200	20	60	135
0	2	200	15	60	187
3	1	150	15	30	48
6	3	200	15	30	80
3	1	200	15	0	45
6	2	200	15	0	64
3	2	200	20	0	81
3	3	200	15	0	82
3	1	200	15	60	82
6	2	200	10	30	62
3	2	200	15	30	88
3	2	200	15	30	89
3	2	200	15	30	91
6	2	200	15	60	85
3	1	250	15	30	69
6	2	200	20	30	92
0	2	200	20	30	180
3	2	250	15	60	114
3	2	200	15	30	93
3	3	200	10	30	84
3	3	250	15	30	105
3	2	250	10	30	85
3	2	250	15	0	75
3	2	150	15	60	86
3	2	150	15	0	45
6	2	150	15	30	54
0	2	150	15	30	120
3	3	200	20	30	120
3	2	150	20	30	92
3	2	150	10	30	69
3	1	200	10	30	46
3	2	200	10	60	99
0	1	200	15	30	106
6	1	200	15	30	54
6	2	250	15	30	87
3	2	200	15	30	90
0	3	200	15	30	165
3	1	200	20	30	85
3	3	200	15	60	120
0	2	200	15	0	107
3	3	150	15	30	83

#### ANOVA

The analysis of variance (ANOVA) is a statistical technique used to test hypotheses. In ANOVA, the primary focus is typically on the “P-Value” column, which determines statistical significance. The P-value is commonly set at 0.05 (a 95% confidence level); a value below 0.05 indicates a significant effect, whereas a value above 0.05 suggests no significant influence. As shown in Table 4, all linear terms (nanoclay, load, speed, time, water soaking), square terms (nanoclay², load², speed², and time²), and interaction terms (nanoclay∗load, nanoclay∗water soaking, time*water soaking) significantly impact mass loss. Generally, the R-square (R²) value should range between 90% and 100% for a model to be considered highly correlated. In this analysis, the R² value is 98.56%, confirming the model’s strong correlation with the experimental data and its overall significance.

**Table 4:** ANOVA table.

Source	DF	Adj SS	Adj MS	F-Value	*P*-Value
Model	20	49969.6	2498.5	85.77	0.000
Linear	5	41385.9	8277.2	284.13	0.000
Nanoclay	1	21535.6	21535.6	739.25	0.000
Load	1	5776.0	5776.0	198.27	0.000
Speed	1	2943.1	2943.1	101.03	0.000
Time	1	3906.2	3906.2	134.09	0.000
Water Soaking	1	7225.0	7225.0	248.01	0.000
Square	5	7192.3	1438.5	49.38	0.000
Nanoclay*Nanoclay	1	4034.2	4034.2	138.48	0.000
Load*Load	1	746.7	746.7	25.63	0.000
Speed*Speed	1	331.9	331.9	11.39	0.002
Time*Time	1	240.5	240.5	8.26	0.008
Water Soaking*Water Soaking	1	4.9	4.9	0.17	0.685
2-Way Interaction	10	1391.5	139.2	4.78	0.001
Nanoclay*Load	1	272.2	272.2	9.35	0.005
Nanoclay*Speed	1	36.0	36.0	1.24	0.277
Nanoclay*Time	1	56.3	56.3	1.93	0.177
Nanoclay*Water Soaking	1	870.3	870.3	29.87	0.000
Load*Speed	1	0.2	0.2	0.01	0.927
Load*Time	1	2.3	2.3	0.08	0.783
Load*Water Soaking	1	0.3	0.3	0.01	0.927
Speed*Time	1	9.0	9.0	0.31	0.583
Speed*Water Soaking	1	1.0	1.0	0.03	0.855
Time*Water Soaking	1	144.0	144.0	4.94	0.035
Error	25	728.3	29.1		
Lack-of-Fit	20	713.5	35.7	12.02	0.006
Pure Error	5	14.8	3.0		
Total	45	50697.9			
**S** − 5.39738 **R-sq** − 98.56% **R-sq(adj)** − 97.41% **R-sq(pred)** − 94.33%					

#### Regression equation and residual plots

Equation (1) presents the full quadratic regression model. The analysis produces R-sq and R-sq(adj) values of 98.56% and 97.41%, respectively. Since these values are very close, they confirm the adequacy of the developed model.

Mass Loss (mg) = -123.7–8.40 Nanoclay + 65.3 Load + 1.228 Speed − 4.53 Time + 0.700 Water Soaking + 2.389 Nanoclay*Nanoclay − 9.25 Load*Load − 0.002467 Speed*Speed + 0.2100 Time*Time − 0.00083 Water Soaking*Water Soaking − 2.750 Nanoclay*Load − 0.0200 Nanoclay*Speed − 0.250 Nanoclay*Time − 0.1639 Nanoclay*Water Soaking + 0.0050 Load*Speed − 0.150 Load*Time + 0.0083 Load*Water Soaking + 0.0060 Speed*Time − 0.00033 Speed*Water Soaking + 0.0400 Time*Water Soaking (1).

**Fig. 3:**
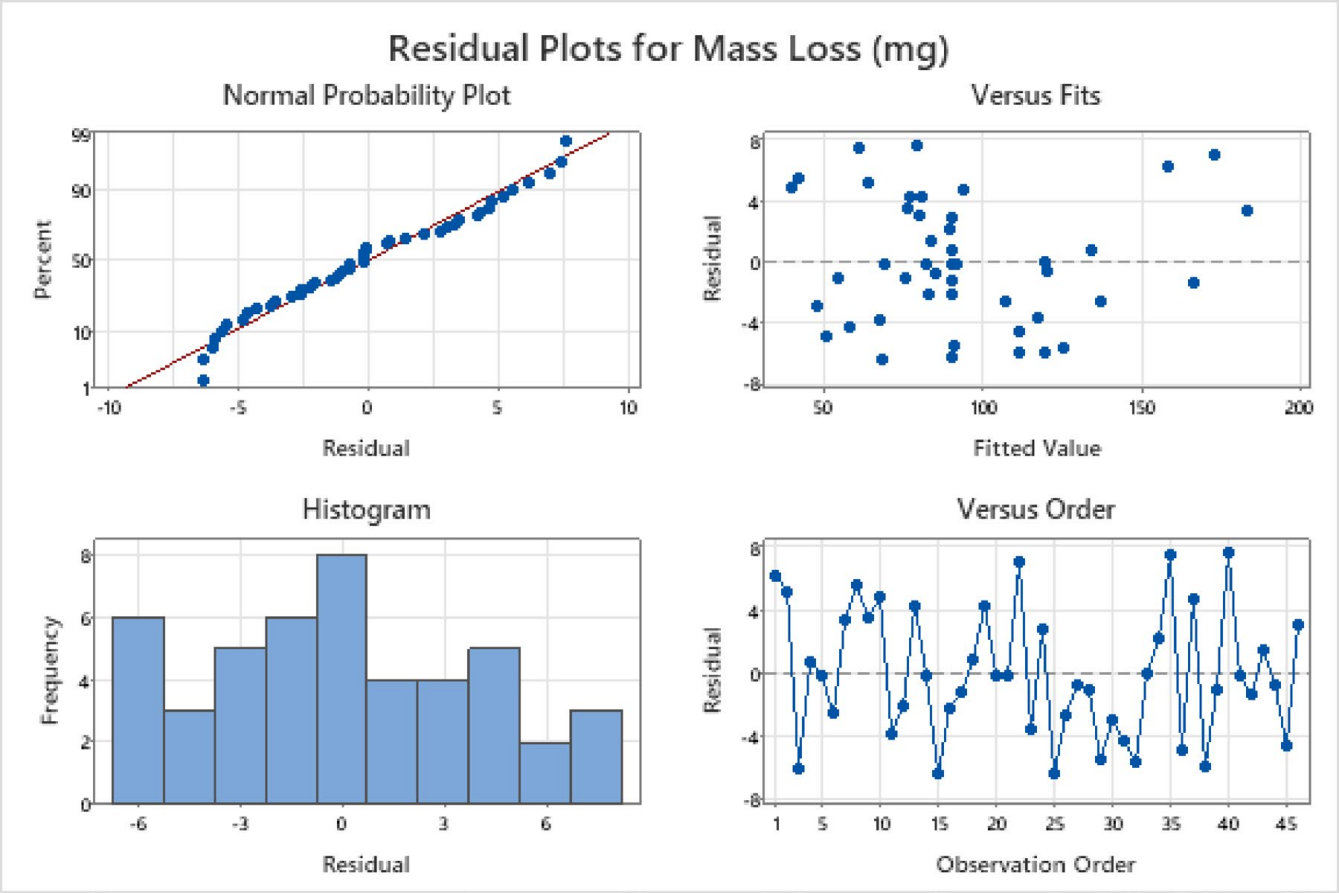
Residual plots for mass loss.

Figure 3 shows that the residuals are closely aligned with the fitted axis, with only minor deviations from a normal distribution. This suggests that the residuals exhibit a normal dispersion, reinforcing a strong linear relationship between the factors and the response variable. In all other graphs, the residuals appear randomly scattered, which is a crucial characteristic for ensuring a good agreement between the experimental and fitted values.

#### Main effect plots

**Fig. 4:**
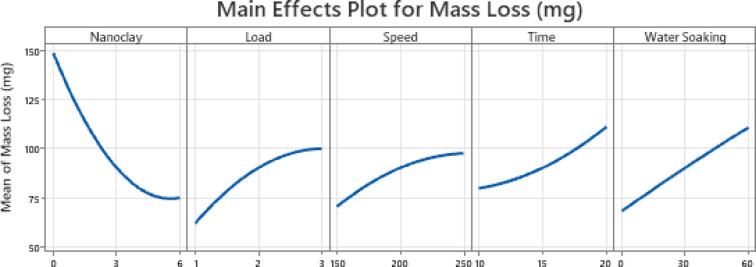
Main effect plot.

The main effects plot (Fig. 4) presents the influence of five parameters, viz., nanoclay wt.%t, load, speed, time, and water soaking, on the wear behavior of the composite material. The wear property is evaluated in terms of mass loss, where higher mass loss indicates greater material degradation due to wear. The trends observed in the plot provide crucial insights into how these parameters affect the wear resistance of the composite.

The plot shows a remarkable decrease in mass loss when nanoclay wt% increases. The pure epoxy (0 wt% nanoclay) samples have a higher mass loss, but incorporating 3 and 6 wt% of nanoclay reduces mass loss. This is because the reinforcing capability of nanoclay improves the mechanical properties of the composite, including hardness and strength. Nanoclay behaves as a wear-resistant barrier for material removal and slows down surface degradation under wear conditions. Nanoclay also plays a role in wear resistance and load-bearing capacity through the prevention of polymer matrix fragment detachment during sliding wear^28–30^.

The mass loss is higher with a rise in applied load, which is a common trend in wear behavior. Increased loads enhance contact pressure and frictional forces at the interface, resulting in increased material removal^31^. Increased load also enhances the development of wear debris, which may further increase surface damage. The rise in wear rate with load is also associated with increased material deformation and microcrack growth in the composite structure^32^.

A direct proportion is seen between speed and mass loss, with wear accelerating with increasing speed. This occurs primarily because frictional heating and surface softening are greater at higher sliding velocities. As speed is raised, more heat is produced at the interface of contact, resulting in thermal degradation of the polymer matrix^33^. In addition, there is a greater transition from the mild to the severe wear state at higher velocities owing to augmented energy dissipation, accelerating the composite surface’s decay^34^.

Mass loss increases with test duration, i.e., material loss increases as the composite is subjected to extended wear conditions. Cumulative surface damage through constant friction and abrasion causes a surface breakdown in the composite structure over time. This indicates that the composite is initially resistant to wear, but increased deterioration is brought about by extended exposure to frictional forces^35^.

Water soaking has a detrimental impact on wear resistance, as indicated by the increase in mass loss with prolonged water exposure. This phenomenon is associated with the moisture absorption behavior of the composite, which leads to hydrothermal aging effects. Additionally, absorbed water can cause plasticization, swelling, and softening of the polymer matrix, reducing its ability to resist frictional forces^10,36^.

#### Interaction plot

**Fig. 5:**
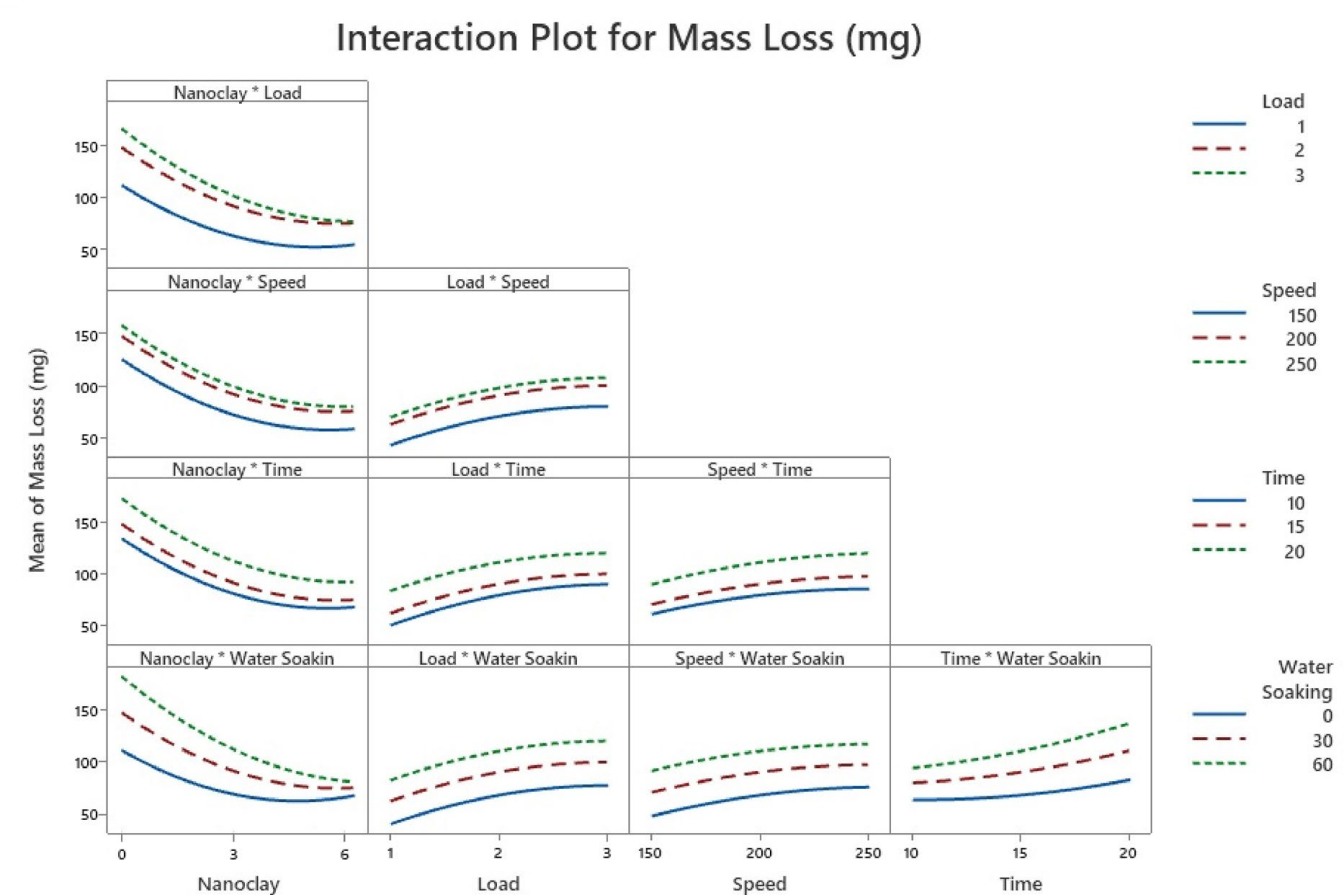
Interaction plot.

Figure 5 depicts the two-way interaction plots, which indicate how distinct input parameters influence each other and their combined effect on mass loss. No interaction among the parameters is displayed if the plot’s lines are parallel. In this study, no interaction between any two components is detected at considered levels. The non-parallel lines between nanoclay∗load and nanoclay∗water soaking suggest potential interaction at higher values, as shown by the P-value of Table 4. Likewise, the lines between time*water soaking are not parallel, showing that lines can interact at lower values, as demonstrated by the P-value. Figure 5 shows that parallel lines between nanoclay*speed, nanoclay∗time, load*speed, load*time, load*water soaking, speed*time, and speed*water soaking indicate no interaction impact (decided by the P-value).

#### Surface plots

**Fig. 6:**
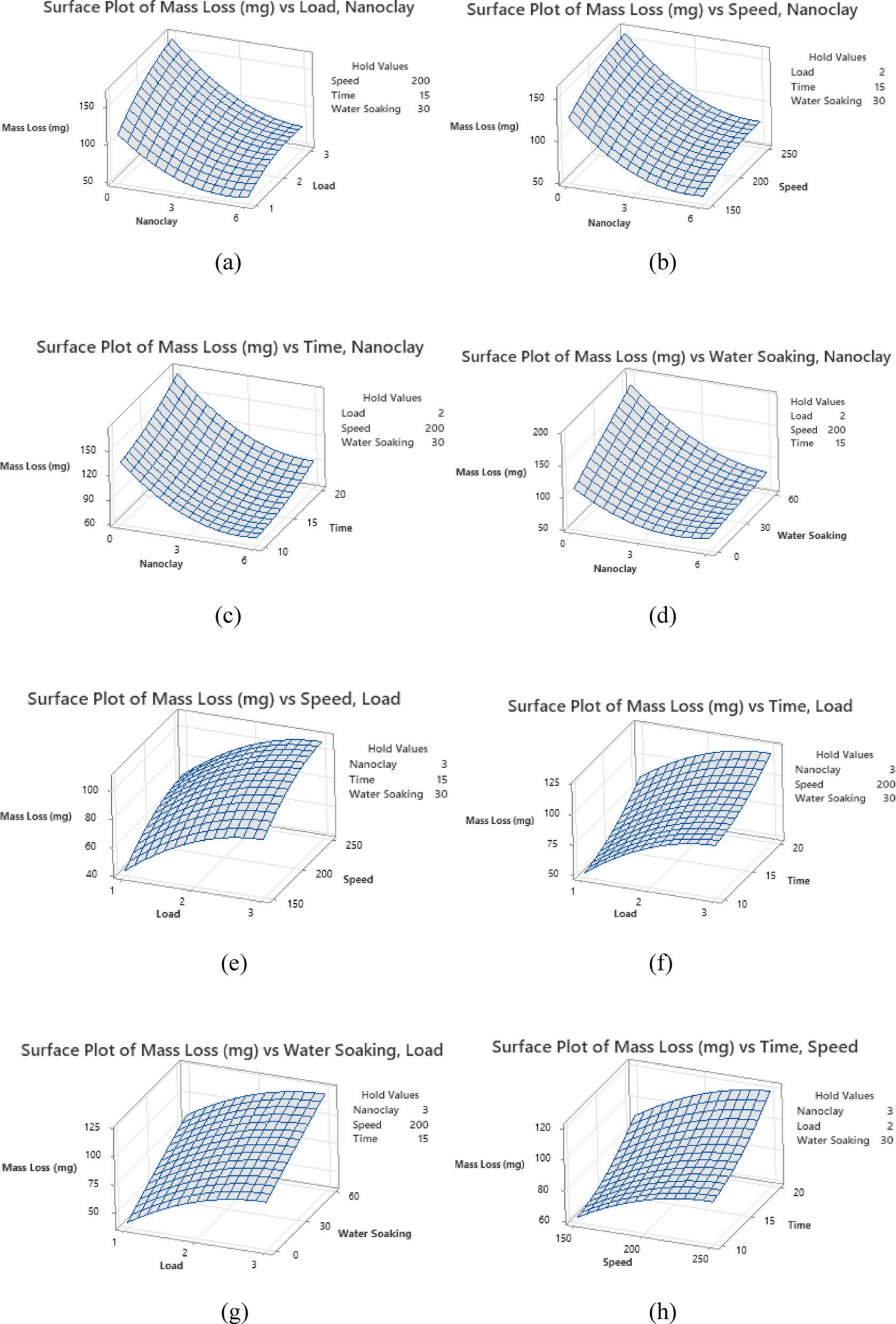

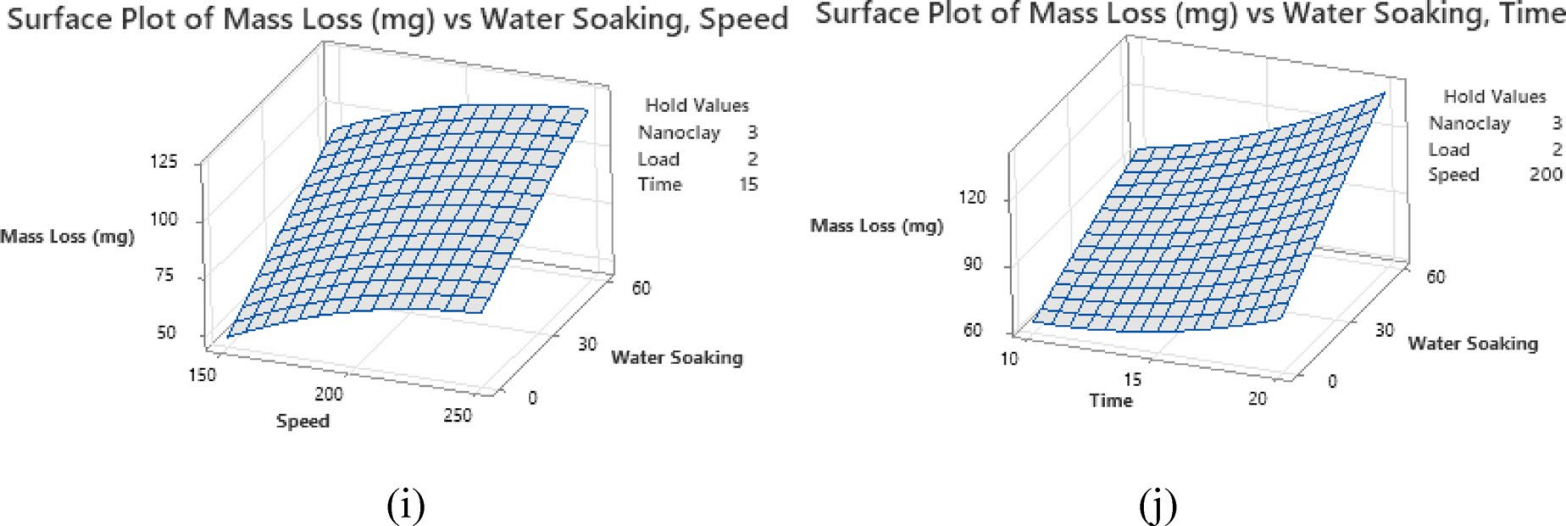
Surface plots.

Figure 6 presents surface plots illustrating mass loss in relation to various factors. In each plot, three factors are kept constant at their mid-levels. The vertical axis represents the mass loss, while the horizontal axes depict the two varying factors. Figure 6 presents the variation in mass loss under different conditions while keeping other factors at their median values. Figure 6(a) shows the relationship between mass loss, applied load, and nanoclay content. Mass loss is highest at 0 wt% nanoclay (pure epoxy) with a 3 kg load and lowest at 6 wt% nanoclay with a 1 kg load. Figure 6(b) illustrates mass loss concerning rotational speed and nanoclay content. The highest mass loss occurs at 0 wt% nanoclay and 250 rpm, whereas the lowest mass loss is observed at 6 wt% nanoclay and 150 rpm. Figure 6(c) depicts mass loss as a function of time and nanoclay content. Mass loss is greatest at 0 wt% nanoclay after 20 min and lowest at 6 wt% nanoclay after 10 min. Figure 6(d) represents the mass loss in relation to water soaking duration and nanoclay content. The highest mass loss is seen at 0 wt% nanoclay after 60 days of water soaking, while the lowest occurs at 6 wt% nanoclay in the dry (as-made) condition. Overall, mass loss increases with higher load, speed, time, and water soaking duration when the nanoclay content remains constant. However, incorporating nanoclay reduces mass loss across all these conditions.

Figure 6 (e) illustrates the influence of speed & load on the mass loss by retaining the other factors at the median level. Similarly, Fig. 6 (f) shows the impact of time & load on the mass loss by maintaining the other factors at the median level. Also, Fig. 6 (g) shows the influence of water soaking & load on the mass loss by maintaining the other factors at the median level. Additionally, Fig. 6 (h) shows the impact of time & speed on the mass loss by retaining the other factors at the median level. Likewise, Fig. 6 (i) confirms the impact of water soaking & speed on the mass loss by retaining the other factors at the median level. At last, Fig. 6 (j) confirms the influence of water soaking & time on the mass loss by retaining the other factors at the median level. Mass loss is high at elevated load, speed, time, and water soaking levels.

### Artificial neural network

**Fig. 7:**
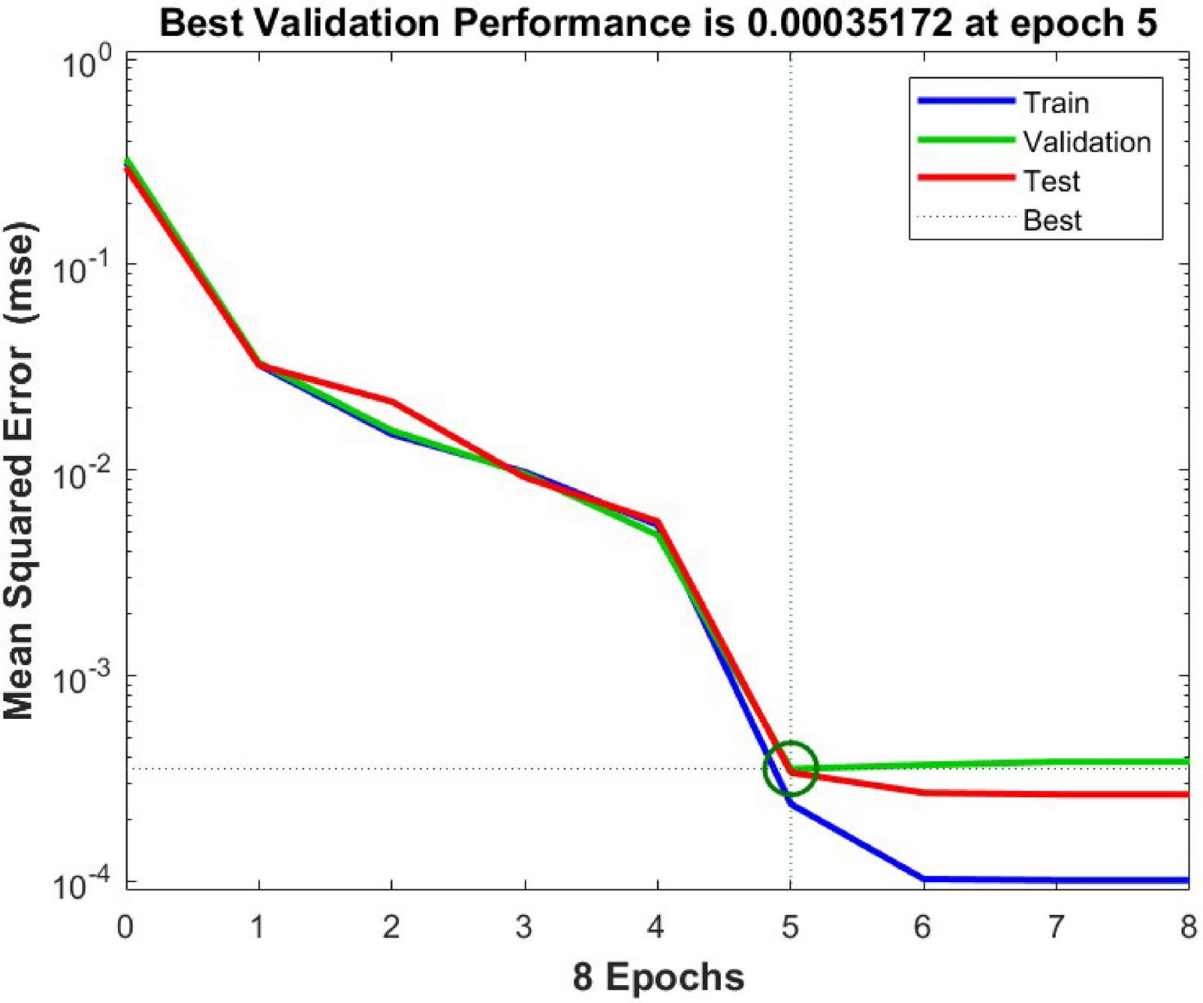
Validation performance curve.

The training and validation loss curve in Fig. 7 illustrates the mean squared error (MSE) over multiple epochs of the training, validation, and test datasets. The training loss consistently decreases, demonstrating that the network is effectively learning from the data. The validation loss initially declines and stabilizes around epoch 5, indicating that the model generalizes well without overfitting. Similarly, the test loss follows the validation loss trend, further confirming the model’s robustness. The best validation performance is achieved at epoch 5, with an MSE of 0.00035172, suggesting that the early stopping criterion is well-tuned and prevents unnecessary overtraining.

**Fig. 8:**
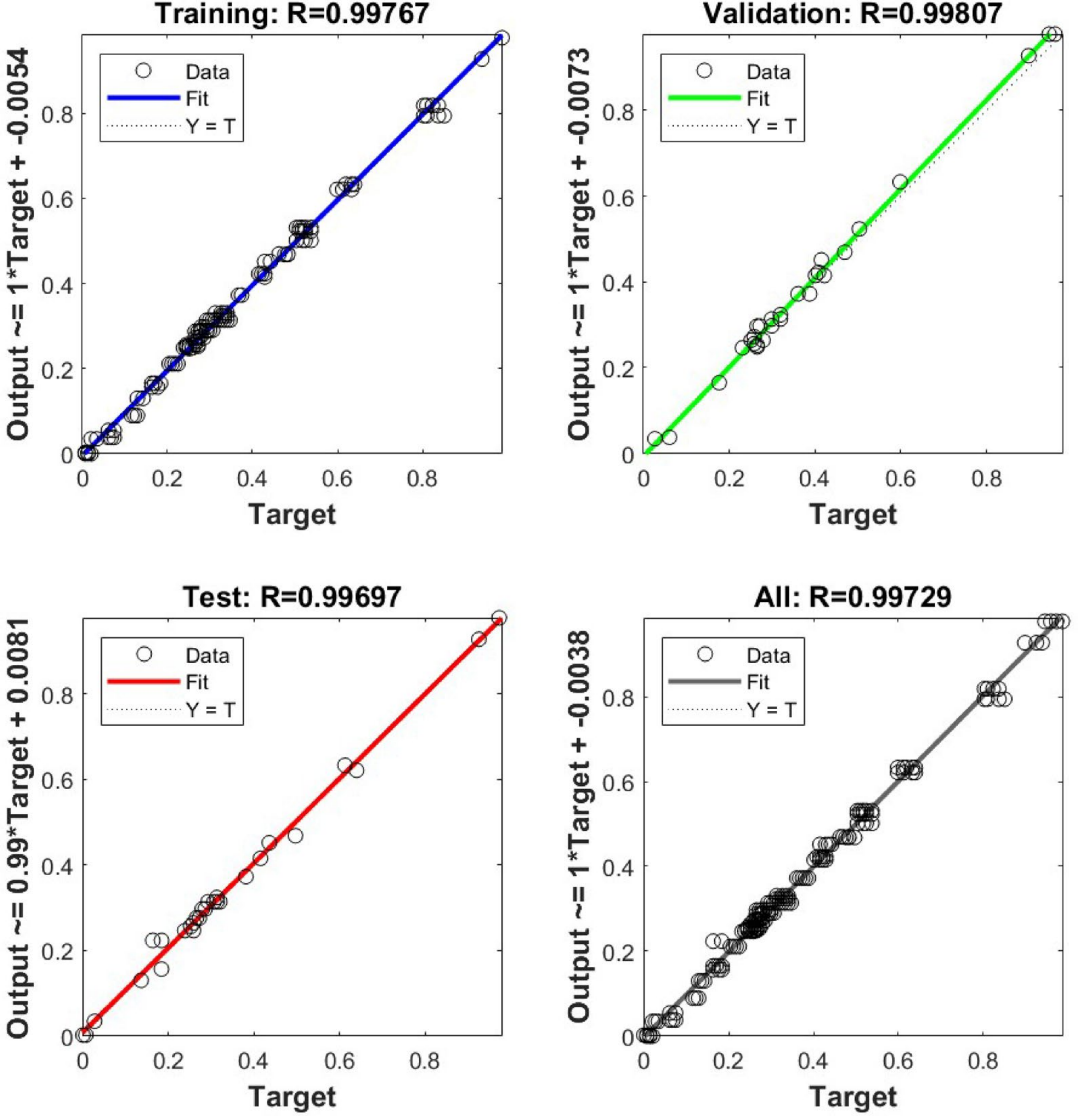
Assessment of ANN model’s performance: regression plots for mass loss.

The regression plots in Fig. 8 show the correlation between the predicted and the actual mass loss values across the different datasets. The training set achieves a correlation coefficient $\:R$ of $\:0.99767$, indicating that the model fits the training data exceptionally well. The validation set, with an $\:R$ value of $\:0.99807,$ suggests excellent generalization to unseen data, while the test set, with $\:R=0.99697$, confirms the model’s reliability in making accurate predictions. The overall $\:R$-value of $\:0.99729$ further reinforces the strong predictive performance. The regression lines closely align with the ideal $\:Y=T$ line, demonstrating a minimal deviation between actual and predicted values.

To further evaluate the model’s goodness of fit, the coefficient of determination $\:\left({R}^{2}\right)$ is computed, yielding a value of 0.99772. This metric quantifies the proportion of variance in actual mass loss values, which the model successfully explains. An $\:{R}^{2}$ a value close to 1 signifies that the ANN effectively captures the underlying patterns in the dataset.

### Comparison data

**Fig. 9:**
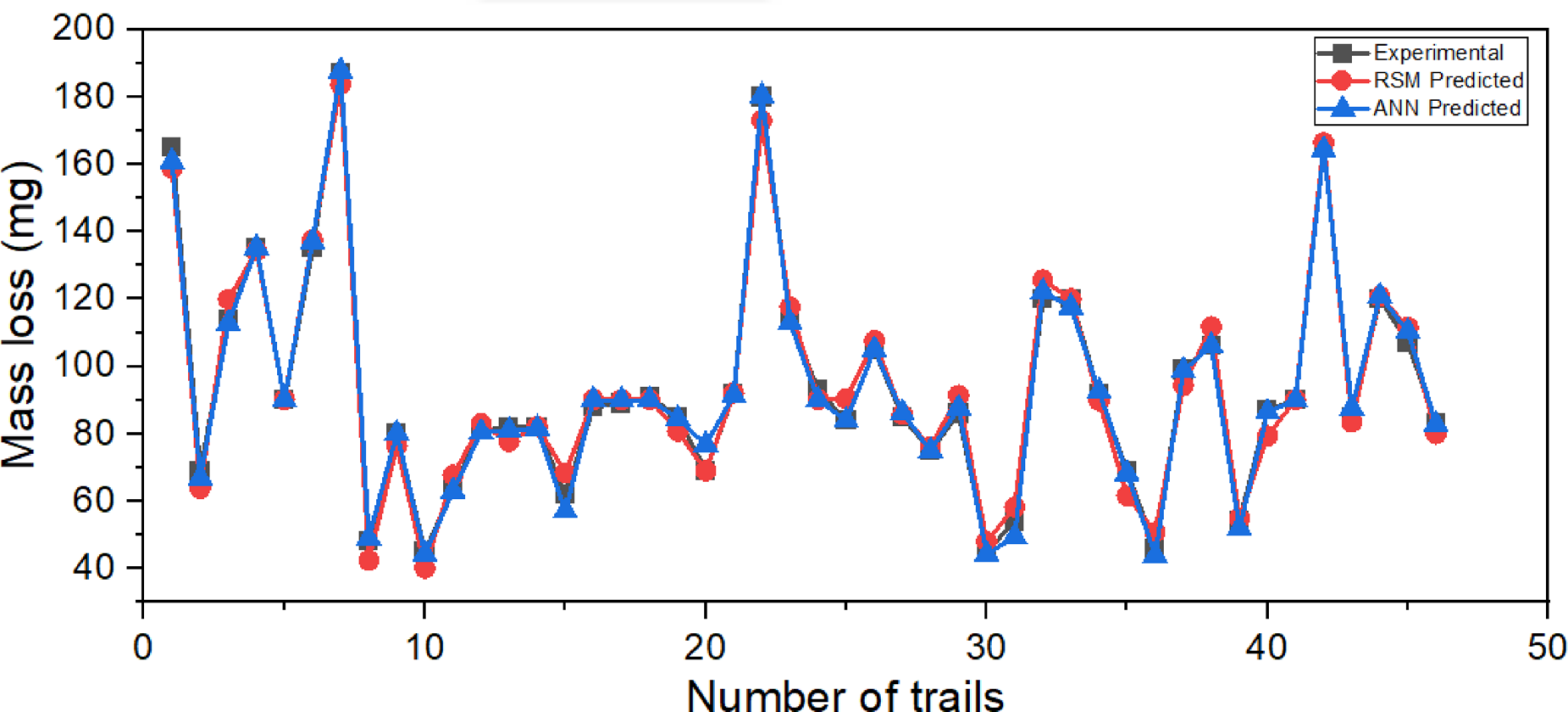
Comparison of experimental findings and the predicted RSM regression values with ANN output.

Figure 9 presents a comparative analysis between the experimental values and the predictions obtained from two modeling approaches: RSM and ANN. The graph plots mass loss (mg) against the number of trials, highlighting the consistency and reliability of the predictive models when compared with actual experimental outcomes.

Figure 9 reveals that both RSM and ANN models are capable of tracking the trend of the experimental data with reasonable accuracy. The general patterns, including fluctuations, peaks, and troughs, are well captured by both predictive approaches. However, it is evident that the ANN model offers a closer fit to the experimental data, particularly in regions with sharp variations in mass loss, such as around trials 1–10, 20, and 40. The ANN’s superior ability to handle nonlinear behavior in complex datasets is likely responsible for this performance advantage. This can be observed from predictive accuracy; the ANN model’s R² value is 0.99772, whereas RSM’s R² value is 98.56%.

The overall tight convergence of the experimental, RSM, and ANN data lines suggests both models’ strong correlation and high predictive reliability. This alignment validates the experimental approach and supports the feasibility of using computational models such as ANN and RSM for simulating mass loss behavior, potentially reducing the need for extensive physical experimentation.

### SEM analysis

**Fig. 10:**
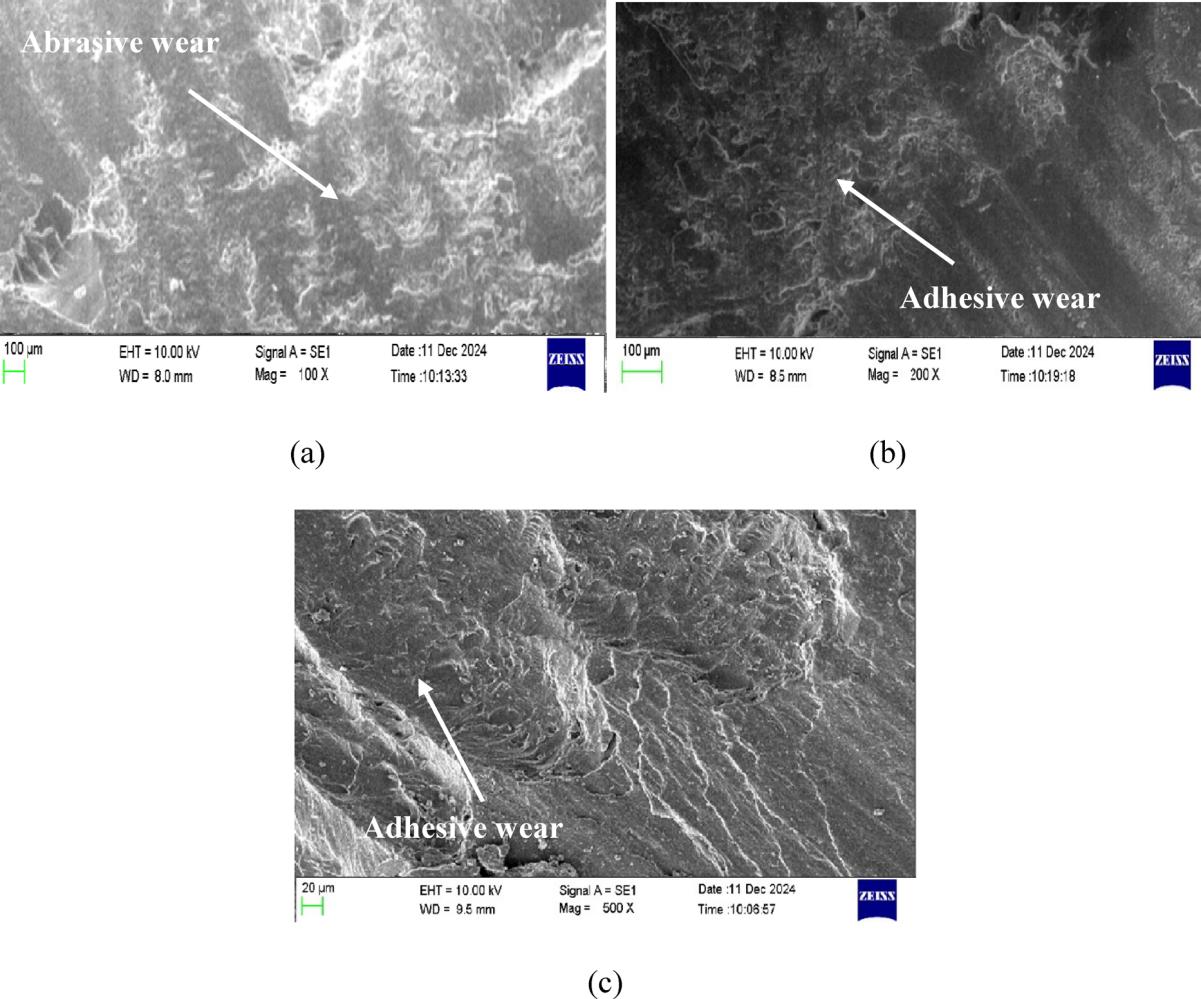
SEM images of the worn surface of pure epoxy.

**Fig. 11:**
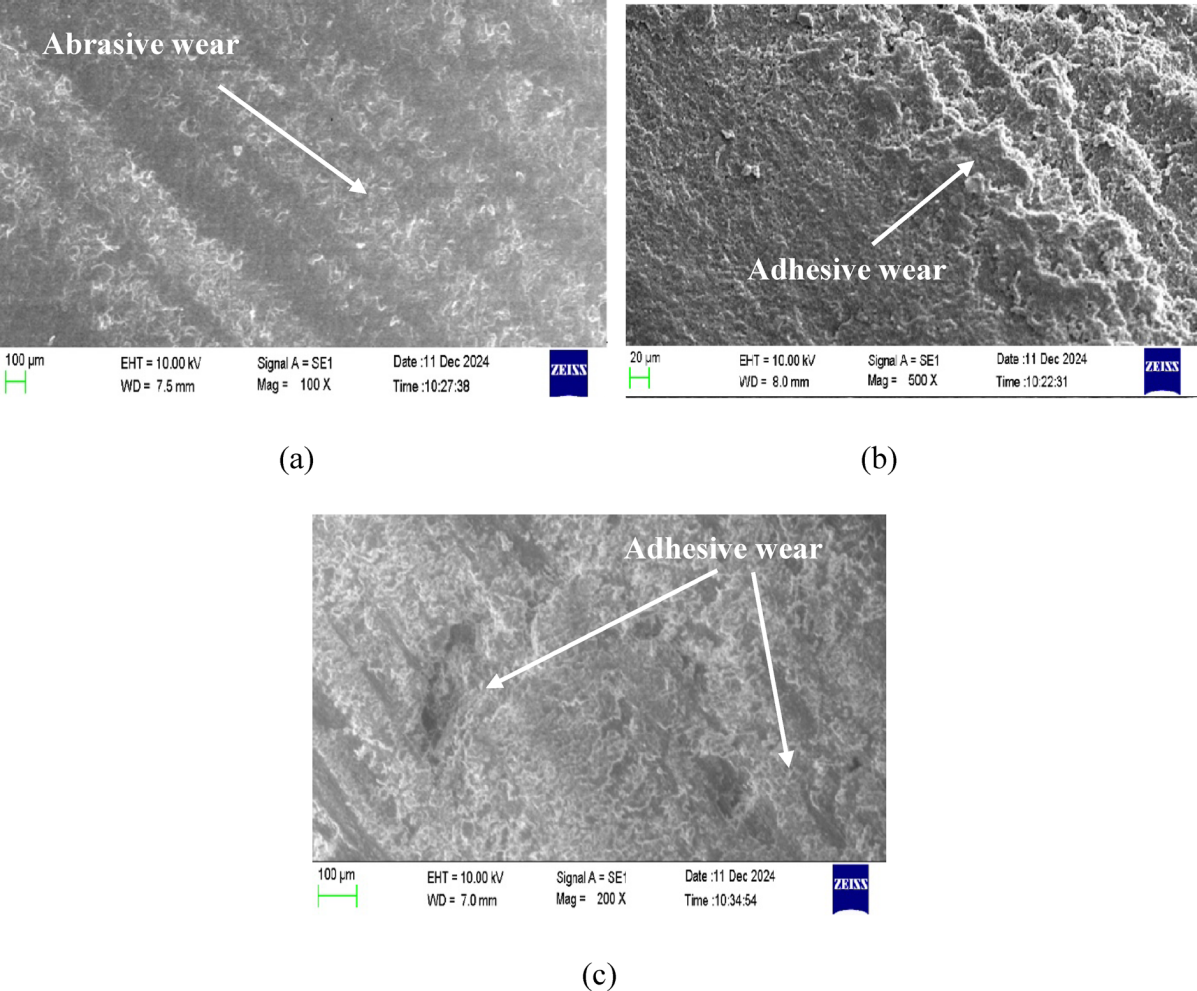
SEM images of the worn surface of 6 wt% of nanoclay-epoxy nanocomposite.

Figures 10 and 11 collectively illustrate the worn surface morphologies of pure epoxy and 6 wt% nanoclay-epoxy nanocomposites under dry conditions and after 30 and 60 days of water soaking. A comparative analysis of these SEM micrographs reveals notable differences in the wear behavior between the pure epoxy and nanoclay-reinforced epoxy systems, offering insight into the role of nanoclay in improving tribological performance, especially in moisture-influenced environments.

In Fig. 10(a), the worn surface of pure epoxy under dry conditions exhibits prominent abrasive wear, evidenced by directional grooves and deep scratches formed by the mechanical action of the rotating disk during sliding. Conversely, Fig. 11(a), which shows the worn surface of the 6 wt% nanoclay-epoxy nanocomposite under similar dry conditions, also reveals abrasive wear features but with shallower and less aggressive grooves. This indicates that incorporating nanoclay enhances the wear resistance by increasing the composite’s hardness and reducing the extent of material removal. The nanoclay particles reinforce the epoxy matrix, making it more resilient against mechanical plowing and micro-cutting.

Under 30 days of water soaking, both materials exhibit a shift in wear mechanisms. Figure 10 (b) (pure epoxy) and Fig. 11 (b) (nanocomposite) show signs of adhesive wear, such as smearing, surface deformation, and localized tearing. However, the pure epoxy surface (Fig. 10 (b)) appears more severely damaged, with extensive material transfer and deeper adhesive wear marks. In contrast, the nanoclay composite (Fig. 11(b)) retains more structural integrity despite showing adhesive characteristics. The presence of nanoclay impedes water ingress and slows down the matrix softening, thus delaying the onset of severe adhesive interactions.

The contrast becomes more pronounced after 60 days of water soaking. Figure 10 (c) reveals intense adhesive wear in pure epoxy, characterized by widespread plastic deformation, flow lines, and tearing. The material appears extensively degraded due to water-induced plasticization and interfacial debonding within the epoxy matrix. On the other hand, Fig. 11 (c) still shows adhesive wear in the nanoclay composite, but the surface degradation is comparatively less severe. Although the wear morphology indicates some interfacial failure and material smearing, the composite structure remains more coherent, suggesting that nanoclay reinforcement improves long-term wear stability even under prolonged moisture exposure.

## Conclusion

- This study comprehensively investigates the wear behavior of nanoclay-epoxy nanocomposites using both RSM and ANN as predictive tools. The research confirms that the addition of nanoclay significantly enhances the wear resistance of epoxy composites by reducing mass loss across various testing conditions. Specifically, the incorporation of 6 wt% nanoclay led to the lowest observed mass loss, attributed to improved load-bearing capacity, reduced surface degradation, and enhanced barrier effects of the nanoclay platelets.
- The statistical model developed through RSM demonstrates a high degree of reliability (R² = 98.56%) in predicting mass loss, with parameters, viz., nanoclay wt%, load, speed, time, and water soaking duration all significantly influencing mass loss. Notably, the ANN model outperformed RSM in predictive accuracy, achieving an R² of 0.99772, underscoring the strength of machine learning approaches for capturing nonlinear interactions in wear analysis.
- SEM analysis reveals distinct wear mechanisms. Worn-out surfaces exhibit both adhesive and abrasive wear with significant material pull-out. Nanoclay-epoxy nanocomposites show smoother wear tracks and reduced damage compared to pure epoxy, validating the protective role of nanoclay.
- Importantly, this work confirms the beneficial effect of nanoclay in enhancing wear resistance and presents a dual-model approach that integrates experimental validation with advanced predictive modeling. The combined use of RSM and ANN offers a robust framework for optimizing composite material performance with minimal experimental trials, contributing a valuable toolset for future material design and engineering applications.

## Data Availability

The data used and/or analysed during the current study is available from the corresponding author upon reasonable request.
